# Benthic biogeographic patterns on the deep Brazilian margin

**DOI:** 10.7717/peerj.14585

**Published:** 2023-02-27

**Authors:** Daniela Y. Gaurisas, Angelo F. Bernardino

**Affiliations:** Departamento de Oceanografia e Ecologia, Universidade Federal do Espírito Santo, Vitória, Espírito Santo, Brazil

**Keywords:** Zoogeography, Deep-sea, Benthos, Distribution, Brazilian EEZ, Water masses, Provinces, Biodiversity, Ecoregions, South Atlantic

## Abstract

The Brazilian continental margin (BCM) extends from the Tropical to the Subtropical Atlantic Ocean, with much of its seafloor within deep waters, supporting rich geomorphological features and under wide productivity gradients. Deep-sea biogeographic boundaries on the BCM have been limited to studies that used water mass and salinity properties of deep-water masses, partly as a result of historical under sampling and a lack of consolidation of available biological and ecological datasets. The aim of this study was to consolidate benthic assemblage datasets and test current oceanographic biogeographical deep-sea boundaries (200–5,000 m) using available faunal distributions. We retrieved over 4,000 benthic data records from open-access databases and used cluster analysis to examine assemblage distributions against the deep-sea biogeographical classification scheme from Watling et al. (2013). Starting from the assumption that vertical and horizontal distribution patterns can vary regionally, we test other schemes incorporating latitudinal and water masses stratification within the Brazilian margin. As expected, the classification scheme based on benthic biodiversity is in overall agreement with the general boundaries proposed by Watling et al. (2013). However, our analysis allowed much refinement in the former boundaries, and here we propose the use of two biogeographic realms, two provinces and seven bathyal ecoregions (200–3,500 m), and three abyssal provinces (>3,500 m) along the BCM. The main driver for these units seems to be latitudinal gradients as well as water mass characteristics such as temperature. Our study provides a significant improvement of benthic biogeographic ranges along the Brazilian continental margin allowing a more detailed recognition of its biodiversity and ecological value, and also supports the needed spatial management for industrial activities occurring in its deep waters.

## Introduction

The deep ocean is the largest ecosystem on Earth, comprising approximately 70% of the global sea-floor area and hence more than half of the planet’s surface (Costello, Cheung & De Hauwere, 2010). This environment encompasses characteristics that make it distinct from other marine and land ecosystems and unique for the entire planet, but, unfortunately, it is also the least explored with many areas still not mapped (Ramirez-Llodra et al., 2010). The South Atlantic Ocean is one of those relatively less explored parts of the global oceans when compared to the North Atlantic, which appears to be the best sampled of all deep-sea environments (Menegotto & Rangel, 2018; Hughes et al., 2021). Although the interest in studying the South Atlantic has increased during the last decades and there has been an increased assessment of its deep-sea ecosystems (Sumida, De Leo & Bernardino, 2020), broad biogeographic patterns of biodiversity and ecosystem functioning remain poorly assessed.

The Brazilian continental margin (BCM) extends along the Tropical and Subtropical Atlantic Ocean (Spalding et al., 2007), and belongs to a tectonically passive-type margin with a typically wide (>100 km) continental shelf. The BCM is within the Brazilian Exclusive Economic Zone (EEZ) that comprises a total area of 3.6 million km^2^, ranking 11th in terms of size worldwide (Sumida, De Leo & Bernardino, 2020; De Leo, Bernardino & Sumida, 2020). It contains an extensive deep-sea territory, with a broad variety of seafloor geomorphological features, which are distributed along wide latitudinal and productivity gradients. The broad range and diversity of seafloor features hosts a great diversity of ecosystems and associated species (De Leo, Bernardino & Sumida, 2020; Magris et al., 2020). Much of these features have been poorly sampled at depths below 200 m, and the Southeast margin has been more sampled and studied due to the interest of the offshore oil and gas industry (Sumida & Pires-Vanin, 1997; Netto, Gallucci & Fonseca, 2005; Bernardino, Berenguer & Ribeiro-Ferreira, 2016; Almada & Bernardino, 2017; Fujikura et al., 2017; Bernardino et al., 2019).

There has been a limited assessment of deep-sea biogeographic ranges along the BCM, which prevents a detailed recognition of its ecological value, its conservation status and threats. For one part, these assessments have lagged ones made over terrestrial ecosystems and coastal waters due to a much-limited sampling (Menegotto & Rangel, 2018; Hughes et al., 2021), and for the other the biogeography realms in the deep ocean have been challenging to define due to a lower taxonomic knowledge of deep-sea species (McFadden et al., 2011). Seminal studies about deep-sea biogeographic patterns focused on a few benthic taxa including asteroids (Sibuet, 1979), tunicates (Monniot, 1979), bivalves (Allen & Sanders, 1996), and cumaceans (Watling, 2009). These studies overall support a division of the South Atlantic in two biogeographical units or provinces segregated primarily by temperature. Nevertheless, detailed latitudinal diversity gradients of species in the deep-sea have been mostly restricted to the North Atlantic (Rex, Stuart & Coyne, 2000; Lambshead et al., 2000; Ellingsen & Gray, 2002).

With evidence that deep-sea species were commonly associated to deep water masses, the early analyses of zoogeographical distributions carried out in deep waters focused mainly on abyssal (Vinogradova, 1979, 1997; Menzies, George & Rowe, 1973) and hadal (Belyaev, 1989) depths. Those were largely based on the topography, origin, and characteristics of the bottom water. However, the detailed works of Zezina (1973, 1976, 1994, 1997) on bathyal brachiopods, opened up further studies on this vast area characterized by its highly heterogeneous physiography and hydrography, and were directly followed by the recognition that deep-sea margins are geologically and biologically diverse (Levin & Sibuet, 2012; Levin & Lebriss, 2020). Zezina (1997) not only proposed a global biogeography for the bathyal depth, but also suggested this zone as a refuge for relict species and living fossils, being comparable to and even surpassing shallow tropical waters and abyssal hydrothermal vents.

Current knowledge suggests that along deep-sea margins geographical boundaries are commonly structured by water masses, temperature and productivity, which are then used to classify species ranges to potential habitat distributions and extension. One of the most relevant biogeographical classifications works is the Marine Ecoregions of the World (MEOW) by Spalding et al. (2007), which proposed both benthic and pelagic realms for the world’s coastal and shelf areas, but defining the lower boundary at 200 m depth. In a more comprehensive attempt, Watling et al. (2013) proposed a biogeography scheme of the deep seafloor based on the concepts regarding regions and provinces previously promoted by Menzies, George & Rowe (1973), Vinogradova (1979), Belyaev (1989), and Zezina (1973, 1997). Their work proposed global “benthic biogeographical units” or provinces for the lower bathyal (801–3,500 m) and abyssal (3,501–6,500 m) depth zones based on water mass characteristics (temperature, salinity, and dissolved oxygen), and included the variability in particulate organic flux (POC) to the seafloor which is key to benthic ecosystem functioning (Smith et al., 2008). However, unlike zoogeographic studies, these provinces remained hypothetical and remain to be tested in many regions with species distribution data. More recently, Summers & Watling (2021) tested the biogeographic scheme for the upper bathyal depths (200–1,000 m) across the Pacific Ocean using octocoral distributions with the MEOW biogeographic scheme and Ecological Marine Units (EMUs) (Sayre et al., 2017), and suggested that shifts from the global biogeographical predictions occur due to regional changes in the geographical species distribution. Although depth, temperature and water masses play important roles, biogeographic depth boundaries can vary by region; which was also previously observed in the North Atlantic (Braga-Henriques et al., 2013).

The limited sampling and integration of deep-sea faunal datasets in the South Atlantic has limited the delineation of its biogeographic realms by oceanographic and water mass parameters. As such, although it is likely that large deep-water provinces in the South Atlantic may be well defined by these parameters, the biological ecoregions or small biogeographical units within these provinces are not defined to date. The delimitation of regional biogeographic areas in the deep South Atlantic could improve species distribution modeling and aid the detection of changes in long-term environmental conditions, as well as the threats to which these assemblages may be exposed due to climate change and anthropogenic activities. These steps are critical for an improved management of Brazil’s deep-sea biodiversity and the definition of ecologically or biologically significant areas (EBSAs). Therefore, our purpose in this study was not only to present the biogeographic patterns of the deep benthic fauna along the BCM, but also, to test two existing biogeographical schemes (Spalding et al., 2007; Watling et al., 2013) for the South Atlantic along bathyal and abyssal depths in the BCM. We used biological datasets from online open access deep-sea benthic distribution records for the Brazilian EEZ. Here we present the first deep-sea biogeographical provinces of the Brazilian continental margin based on benthic species distribution from bathyal to abyssal depths, and discuss its relationship with deep-water masses within the Brazilian EEZ.

## Materials and Methods

### Study area

**Figure 1:**
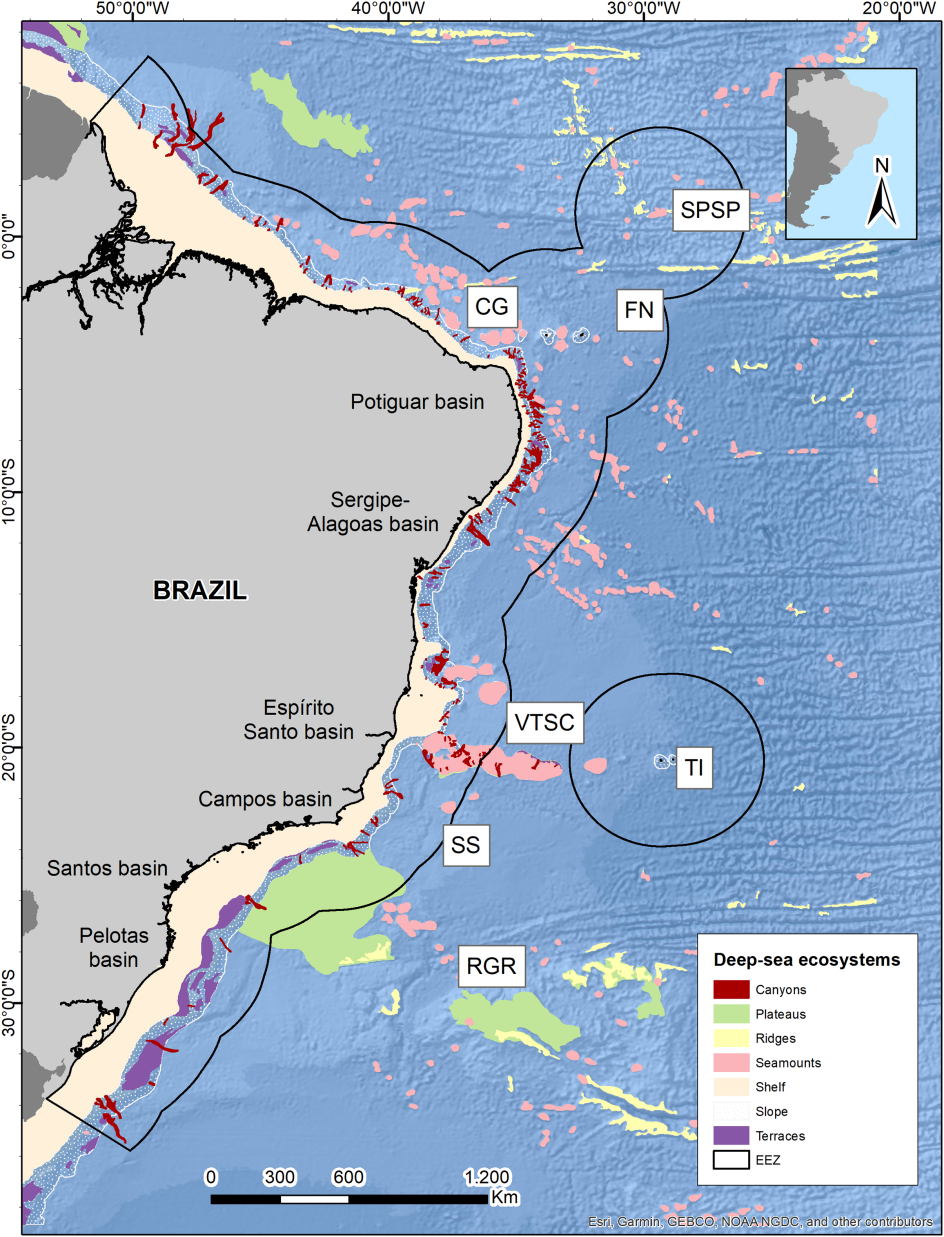
Brazilian continental margin with deep-sea ecosystems. Main sedimentary basins along the margin are indicated. Brazil’s EEZ delimited within the black line. Seamounts and oceanic islands abbreviations: SPSP, St Peter St Paul; CG, Ceará guyot; FN, Fernando de Noronha Archipelago; VTSC, Vitória-Trindade seamount chain; TI, Trindade Island; SS, Saldanha seamount; RGR, Rio Grande Rise. Deep-sea ecosystems GIS database from Blue Habitats (UNEP). Data credit: Esri, Garmin, NOAA NGDC, and other contributors.

The Brazilian EEZ covers a total area of 3,642,070 km^2^, which extends through the tropical and subtropical Southwestern Atlantic Ocean. It contains an extensive deep-sea territory with a wide variety of seafloor features distributed along latitudinal and productivity gradients. One of the largest and most notorious geomorphological features is the Vitoria-Trindade Ridge (VTR; 21°S), a seamount chain adjacent to the BCM that naturally delimits the tropical and subtropical portions of the BCM and the contiguous abyssal plains (De Leo, Bernardino & Sumida, 2020) (Fig. 1).

**Figure 2:**
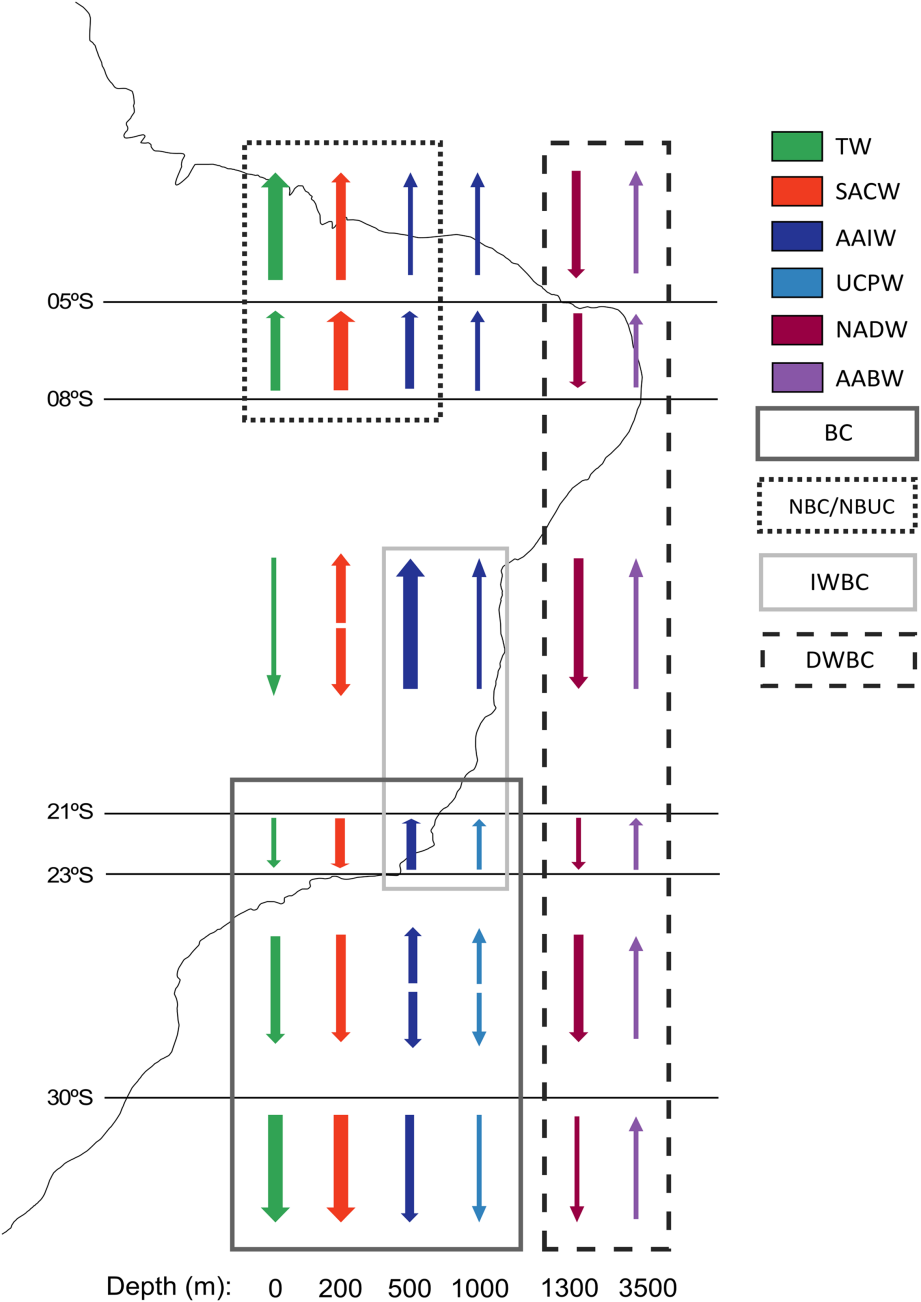
Primary currents and water masses along the Brazilian Continental Margin. Arrows represent the direction of the current and their thickness represents the relative current intensity. The water masses transported by each current are marked inside a rectangle. Water masses abbreviations: TW, Tropical Water; SACW, South Atlantic Central Water; AAIW, Antarctic Intermediate Water; UCPW, Upper Circumpolar Water; NADW, North Atlantic Deep Water; AABW, Antarctic Bottom Water. Currents abbreviations: BC, Brazil Current; DWBC, Deep Western Boundary Current; IWBC, Intermediate Western Boundary Current; NBC, North Brazil Current; NBUC, North Brazil Undercurrent (Modified from Sumida, De Leo & Bernardino, 2020).

The vertical distribution of water masses in the Southwestern Atlantic Ocean exhibits variations along the South American continental border. There are six primary water masses composing the water column in the Southwestern Atlantic, and five of them correspond to deep waters: South Atlantic Central Water (SACW), Antarctic Intermediate Water (AAIW), Upper Circumpolar Water (UCPW), North Atlantic Deep Water (NADW), and the Antarctic Bottom Water (AABW) (Silveira et al., 2015) (Fig. 2). Each water mass bathes different depths and latitudinal ranges over the BCM and adjacent abyssal plain, exhibiting different physical and chemical properties. Thus, the tropical deep-water column in the Brazilian Continental Margin is composed of SACW, AAIW, NADW, and AABW, differing from the subtropical by the absence of UCPW, which has been identified by an oxygen minimum associated with a local silicate maximum (Mémery et al., 2000), and its northern limit surrounds the Vitória-Trindade Seamounts (Tsuchiya, Talley & McCartney, 1994) (Fig. 2).

These water masses are transported by three principal western boundary current systems. The subtropical Brazil Current (BC) and the tropical North Brazil Current (NBC) correspond to main upper-ocean current systems occurring along the BCM. As the upper-ocean contains the mixed layer, the seasonal pycnocline, and the permanent pycnocline, these currents are responsible for transporting surface (TW), central (SACW), and intermediate (AAIW and UCPW) water masses (Silveira, Napolitano & Farias, 2020). Below the permanent pycnocline (1,000–1,300 m depth), the NADW is carried south by the Deep Western Boundary Current (DWBC), which constitutes the main component of the Atlantic Meridional Overturning Circulation (AMOC). On the other hand, as the intermediate response to the AMOC, the Intermediate Western Boundary Current (IWBC) flows equatorward carrying the intermediate waters. Finally, the AABW is not associated with any western boundary current unlike all other water masses over the BCM. Bottom water extends over the entire South Atlantic seabed, limited by the topography of the abyssal plain (Silveira, Napolitano & Farias, 2020) (Figs. 1 and 2).

### Benthic dataset

Existing and openly published databases of deep-sea benthos were obtained in order to synthesize the deep-sea benthic diversity from the Brazilian EEZ. These databases are available online in OBIS (Ocean Biodiversity Information System) and from some relevant natural marine history museums and studies carried out in Brazilian deep basins. The final database was curated and standardized in order to avoid discrepancies and errors. Records outside the EEZ were removed from the database, as well as those that were not identified to species level. The taxonomic status of all records was revised and updated following the World Register of Marine Species site (WoRMS Editorial Board, 2021).

Species occurrence mapping was carried out in ArcGIS 10.5 software (ESRI, 2016), creating a shapefile for each benthic phylum, delimitating the distribution to only deep-water records (200–5,000 m), using the GEBCO bathymetry (GEBCO Compilation Group, 2021). Several relevant deep-sea ecosystems in the Brazilian EEZ were mapped using the global seafloor geomorphic features map (GSFM) (Harris et al., 2014), which follows the seabed geomorphic features defined by the International Hydrographic Organization (IHO). The final database as well as the distribution shapefiles are publicly available at Gaurisas & Bernardino (2022).

### Biogeographical classification schemes

**Figure 3:**
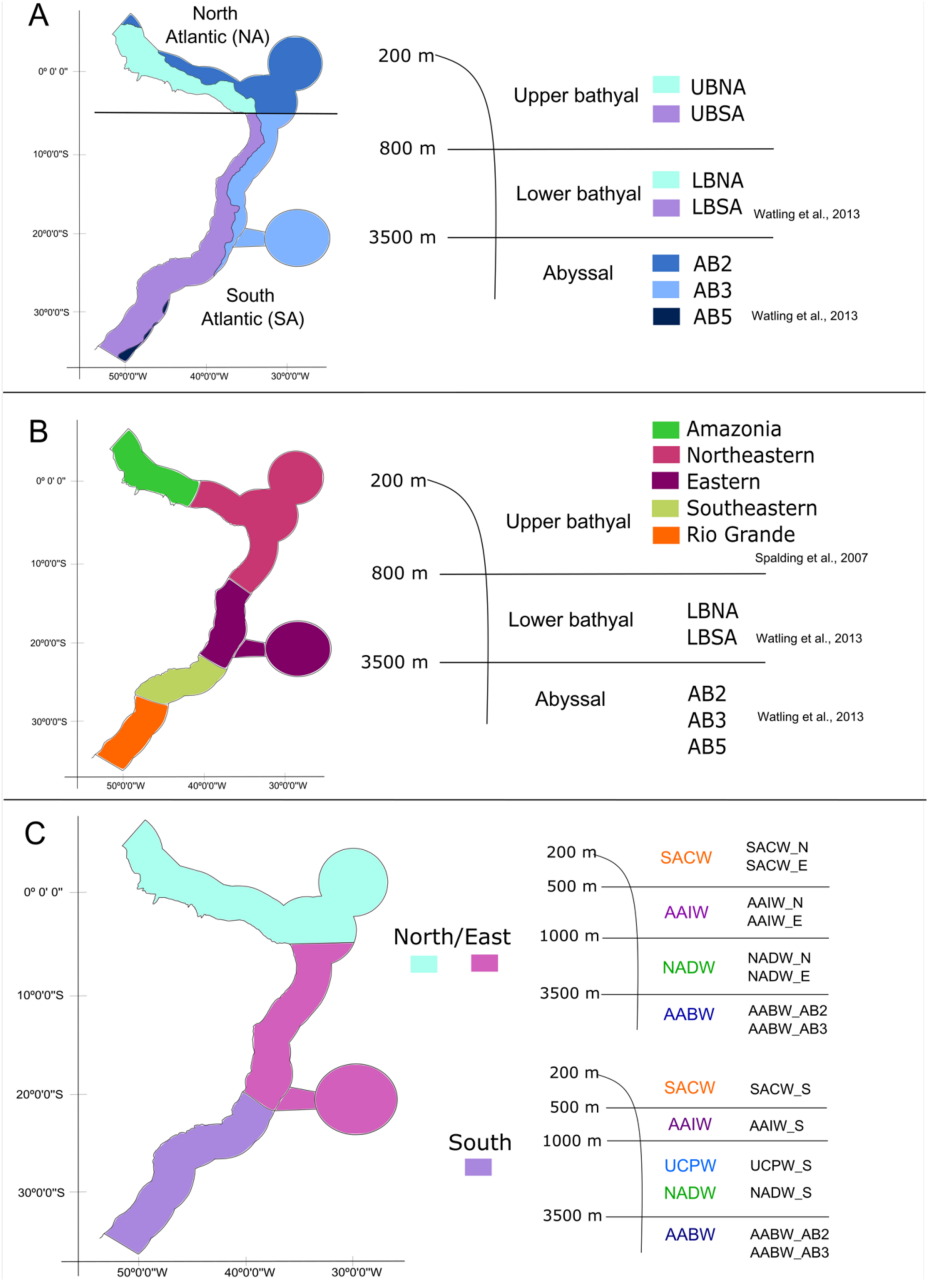
The three different biogeographic schemes or scenarios tested in the present work. (A) The Watling et al. (2013) deep-sea biogeographic scheme. Bathyal provinces: UBNA, Upper Bathyal North Atlantic; UBSA, Upper Bathyal South Atlantic; LBNA, Lower Bathyal North Atlantic; LBSA, Lower Bathyal South Atlantic, Abyssal provinces: AB2-North Atlantic, AB3-Brazil Basin, AB5-Argentine Basin; (B) a hybrid classification of depth and marine ecoregions following Spalding et al. (2007) and Watling et al. (2013); (C) the Brazilian deep-water masses biogeographic scheme. Water masses: SACW, South Atlantic Central Water; AAIW, Antarctic Intermediate Water; UCPW, Upper Circumpolar Water; NADW, North Atlantic Deep Water; AABW, Antarctic Bottom Water.

With the database in hand, we tested three schemes of biogeographic realms for the Brazilian deep-sea EEZ. In the first one, the definition and delimitation of the deep-sea provinces for the BCM was based on the deep-sea biogeography proposed by Watling et al. (2013), which has three depth ranges: upper bathyal (200–800 m), lower bathyal (801–3,500 m; two provinces) and abyssal (3,501–6,500 m; three provinces). Given that the authors did not determine the upper bathyal provinces in their work, we defined two upper bathyal provinces for the Brazilian EEZ between 200–800 m that were named as the North Atlantic Upper Bathyal (UBNA) and the South Atlantic Upper Bathyal (UBSA) following the same boundaries of the lower bathyal provinces. The two lower bathyal provinces from Watling et al. (2013) (North Atlantic Bathyal and South Atlantic) were named here as North Atlantic lower bathyal (LBNA) and South Atlantic lower bathyal (LBSA), respectively, for better understanding. The nomenclature for the abyssal zone was similar to the existing classification: North Atlantic (AB2), Brazil Basin (AB3), and Argentine Basin (AB5) (Fig. 3A).

Considering that our area of interest is the Brazilian EEZ, and that the Watling et al. (2013) scheme had a wider scope, in the second biogeographic scheme we compared our dataset with the Marine Ecosystems of the World classification (Spalding et al., 2007) along upper bathyal depths and using Watling et al. (2013) classification of lower bathyal and abyssal provinces as they are absent from the MEOWs analysis (Fig. 3B). Therefore, five ecoregions or small biogeographical units were considered from the MEOW: Amazonia, Northeastern (including oceanic islands of Fernando de Noronha and Atoll das Rocas), Eastern (including Trindade and Martim Vaz Islands), Southeastern, and Rio Grande (Fig. 3B).

In a third biogeographic scheme, we tested the distribution of the deep-sea benthic fauna along depth by comparing the limits of the deep-water masses present on the deep Brazilian margin. In this case, we divided the bathyal zone of the BCM into three regions (North, East, and South) based on the currents and water masses distribution and their latitudinal range. The Watling et al. (2013) south boundary for the north region (5°S) was maintained, thereafter, the East region was delimited to the south at about 21°S by the Vitória-Trindade Ridge, which marks the tropical and subtropical portions of the BCM and is the northern limit of the UCPW water mass (Fig. 3C). The North, East and South regions were divided into the water masses (SACW, AAIW, UCPW, NADW) and the benthic species records were grouped according to the latitudinal and depth distribution in each of the 10 bathyal biogeographical units (200 to 3,500 m, Fig. 3C). In the abyssal zone, the Watling et al. (2013) provinces for the Atlantic Ocean were maintained, given that only the AABW water mass (>3,500 m depth) is present at that depth along the entire Brazilian margin (Fig. 3C).

### Data analysis

Deep-sea benthic occurrence records were assigned to the biogeographical units they were within and were plotted over the biogeographical units for each scheme using ArcGIS 10.5 software (ESRI, 2016). The biogeographical units with no occurrences were removed from the analysis. All species occurrence data were transformed to presence/absence to reduce the biases in sampling methods and effort across regions.

We used the Simpson’ dissimilarity index to construct dissimilarity matrices based on the occurrence data for each biogeographic scheme (Simpson, 1960). Simpson’s index is based on the spatial turnover of species and is thus less influenced by differences in species richness among regions (Baselga, 2012; Castro-Insua, Gómez-Rodríguez & Baselga, 2018). We then performed cluster analyses on this matrix of dissimilarities. All the analyses were carried out in software R version 4.1.0 (R Core Team, 2021) by using the ‘recluster’ package (Dapporto et al., 2020), where the ‘simp.dist’ function was used to construct the dissimilarity matrices of turnover for faunistic beta-diversity.

It has been recently shown that the topology of hierarchical cluster dendrograms is affected by the order of rows and columns in dissimilarity matrices produced by beta-diversity turnover (Dapporto et al., 2013). That is, the computation of the Simpson dissimilarity index on the original presence/absence matrix can result in many tied values (*i.e*., many spatial units may have identical values of Simpson dissimilarity index, particularly when equal zero, which denotes that there is no species replacement between two areas) (Dapporto et al., 2013). This generates a lot of equivalent and different clustering solutions for the same dissimilarity matrix. To avoid it, recent literature has suggested resampling the original order of the dissimilarity matrix to generate a number of dendrograms and then calculate a consensus tree based on a 50% consensus for any cluster (Dapporto et al., 2013). Following Dapporto et al. (2013), we first performed hierarchical cluster analyses using the unweighted pair group algorithm (UPGMA), which facilitates regionalization by converting dissimilarity matrices into dendrograms using the original order of sites (Kreft & Jetz, 2010; Dapporto et al., 2013). Next, to avoid the possible inconsistent topologies resulting from the fraction of zero and tied values and to provide bootstrap support, we produced first consensus trees after randomly reordering the rows in the dissimilarity matrices with 1,000 resampled trees and a 50% node consensus rule (‘recluster.cons’ function). To support the nodes, a successive bootstrap analysis was conducted by constructing 1,000 consensus trees each made up of 100 reordered sites (‘recluster.boot’ function).

To give each species the opportunity to be included in the bootstrap matrices, and strengthen the support for nodes (Dapporto et al., 2013), we performed resampling using multiscale bootstrap analysis with the same ‘recluster.boot’ parameters and 10 levels (‘recluster.multi’ function). Finally, we inspected the trend of support at different scales by using the ‘recluster.identify.nodes’ function. The values of the nodes on the final ultrametric trees correspond to percentages of times that each node is repeated between different consensus rules; this makes it possible to recognize which links between biogeographical units are supported by data and which are uncertain (Dapporto et al., 2013). The results of the three analyzed biogeographic schemes were compared.

## Results

**Figure 4:**
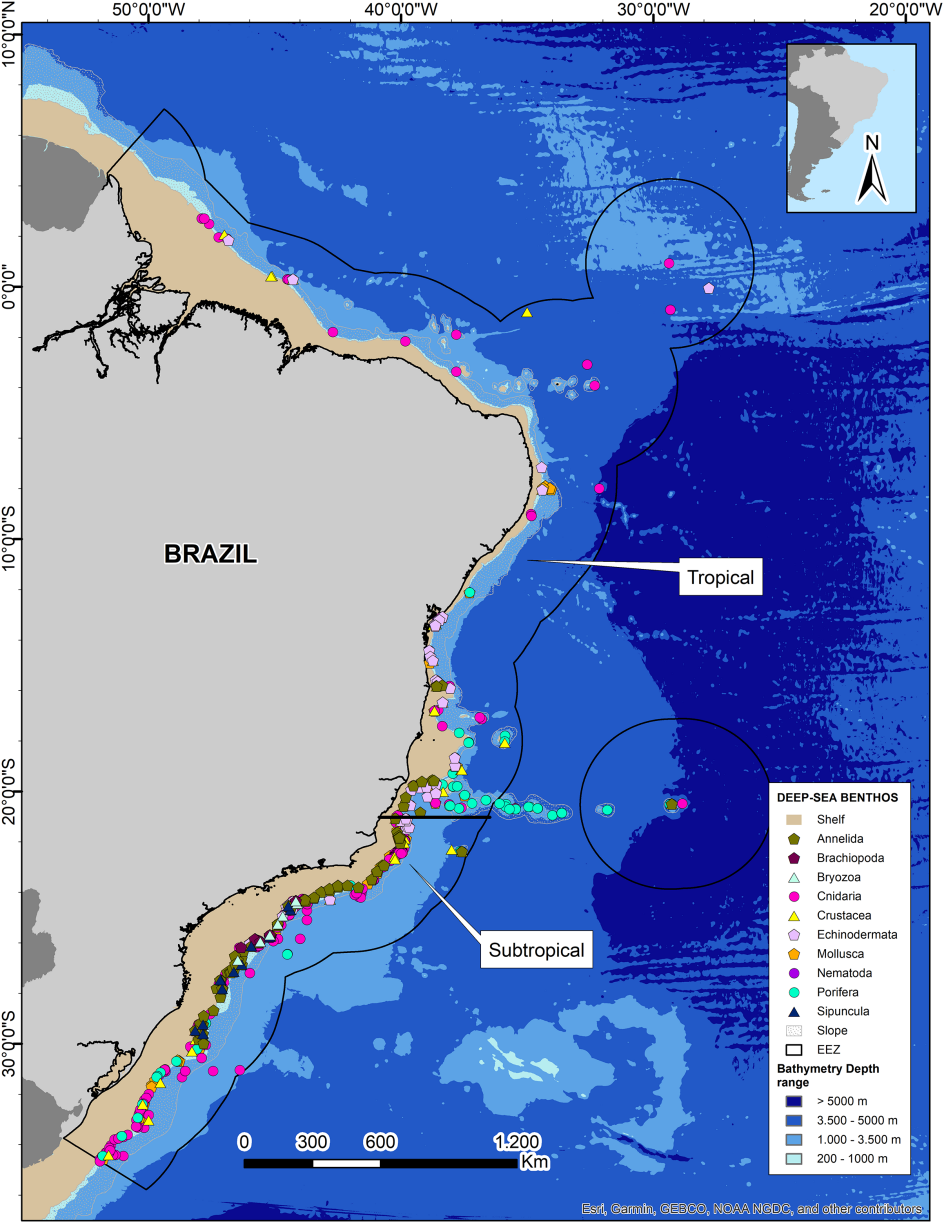
Deep-sea benthos taxa distributed along the Brazilian deep-sea EEZ. The tropical and subtropical regions of the Brazilian continental margin are highlighted with white rectangles. Data credit: Esri, Garmin, NOAA NGDC, and other contributors.

We obtained 14 datasets from the OBIS database in addition to records from the Smithsonian National Museum of Natural History (NMNH) and Universidade Federal do Espírito Santo (UFES). After data curation and standardization, we obtained a total of 4,167 benthic Metazoan occurrence records within the deep Brazilian EEZ (Table 1). Mapping of the records showed that 97% are distributed at bathyal depths, being mostly concentrated at depths less than 1,000 m, and only ten records at abyssal depths >3,000 m. The greatest richness and number of records is reported from the subtropical region of Brazil (67%; Fig. 4).

**Table 1:** Number of deep-sea benthic occurrences and species per phyla distributed throughout the Brazilian EEZ.

Phylum	Tropical Brazilian		Subtropical Brazilian	
	# Occurrences	# Species	# Occurrences	# Species
Annelida (Polychaeta)	0	0	695	191
Arthropoda (Crustacea)	4	3	317	162
Brachiopoda	0	0	10	3
Bryozoa	0	0	17	8
Cnidaria	46	32	1,705	121
Echinodermata	13	10	277	72
Mollusca	19	15	717	314
Nematoda	0	0	49	26
Porifera	0	0	273	74
Sipuncula	0	0	25	7
	**82**	**60**	**4,085**	**978**

**Note:**

* The total values of deep-sea benthic occurrences and species by Brazilian region are in bold.

We retrieved a total of 1,022 species grouped into 10 phyla, with Mollusca and Annelida showing the highest species richness with 326 and 191 species respectively. However, Cnidaria has the highest number of records (1,751) belonging to 142 species (Table 1). The tropical region of Brazil showed a lower number of records and species richness, with less than 50 records for a few phyla and no records for some common and well-known phyla such as Annelida and Porifera (Table 1). On the other hand, the Brazilian Subtropical region had representatives from ten phyla and the greatest richness, but some phyla such as Sipuncula, Brachiopoda, Bryozoa, and Nematoda remained underrepresented (Table 1, Fig. 4). The species with highest number of records were the cold-water corals (CWCs) *Enallopsammia rostrata*, *Lophelia pertusa* (=*Desmophyllum pertusum*), *Madrepora oculata*, and *Solenosmilia variabilis* in bathyal depths of both subtropical and tropical regions. The polychaeta genera *Kinbergonuphis* was among the most reported in the Subtropical region, as well as the sponge genera *Aplysina* and the ophiuroid *Ophiomisidium tommasii*.

### Biogeographical scheme 1

#### The Watling et al. (2013) deep-sea biogeographic scheme

**Figure 5:**
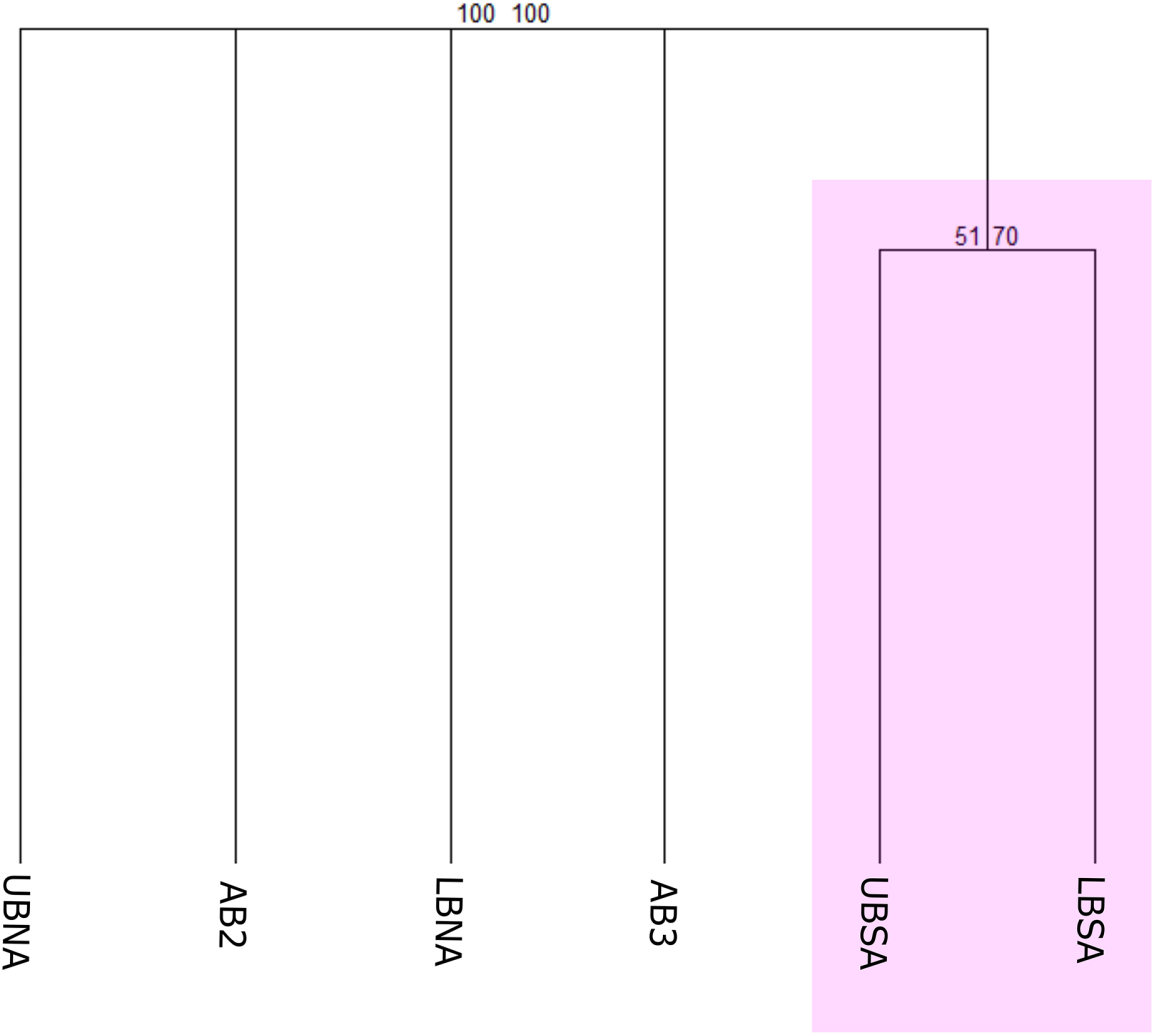
Consensus tree exhibiting the similarity between the deep Atlantic provinces proposed by Watling et al. (2013) in terms of their benthic fauna. The consensus tree obtained by resampling (1000×) the row order and a 50% node consensus rule for faunistic beta-diversity computed with the ‘recluster’ package (Dapporto et al., 2020) is represented together with bootstrap (BP) values for two different scales of bootstrap: X1 (left) and X3 (right). Nodes with a considerable increase in support are shown in black and nodes without a constant increase in red. Provinces abbreviations: UBNA, Upper bathyal North Atlantic; UBS, Upper bathyal South Atlantic; LBNA, Lower bathyal North Atlantic; LBSA, Lower bathyal South Atlantic; AB2, Abyssal North Atlantic; AB3, Abyssal Brazil Basin. Clusters with strong bootstrap node support are highlighted in different colors.

The first biogeographical scheme tested compared the benthic species occurrences according to Watling et al. (2013) provinces (North Atlantic Lower Bathyal (LBNA), South Atlantic Lower Bathyal (LBSA), North Atlantic (AB2), Brazil Basin (AB3), and Argentine Basin (AB5)) and the proposed provinces for the Upper Bathyal (North Atlantic Upper Bathyal (UBNA), South Atlantic Upper Bathyal (UBSA)) (Fig. 3A). The resulting cluster of deep-sea benthic records showed a clear division between the bathyal depths from North and Southern regions (Fig. 5). In the Northern region, the Upper and Lower bathyal ecoregions seem strongly dissimilar from each other, whereas benthic assemblages were more similar across depth in the Southern region, forming a cluster with good bootstrap node support. There, corals of the genera *Acanella*, *Deltocyathus*, and *Trochocyathus* (in addition to the four CWCs mentioned above), and mollusks of the genera *Acteon*, *Benthonella*, and *Brookula* appear as shared species between the upper and lower bathyal provinces. In the North region, UBNA and LBNA appear as individual biogeographical units, with only *Lophelia pertusa* (=*Desmophyllum pertusum*) as a shared species. The Abyssal provinces appear also as individual units with no resemblance to any other province or to each other (Fig. 5). We do not have data for province AB5, so it was excluded from the analysis.

### Biogeographical scheme 2

#### A hybrid classification with depth and marine ecoregions

**Figure 6:**
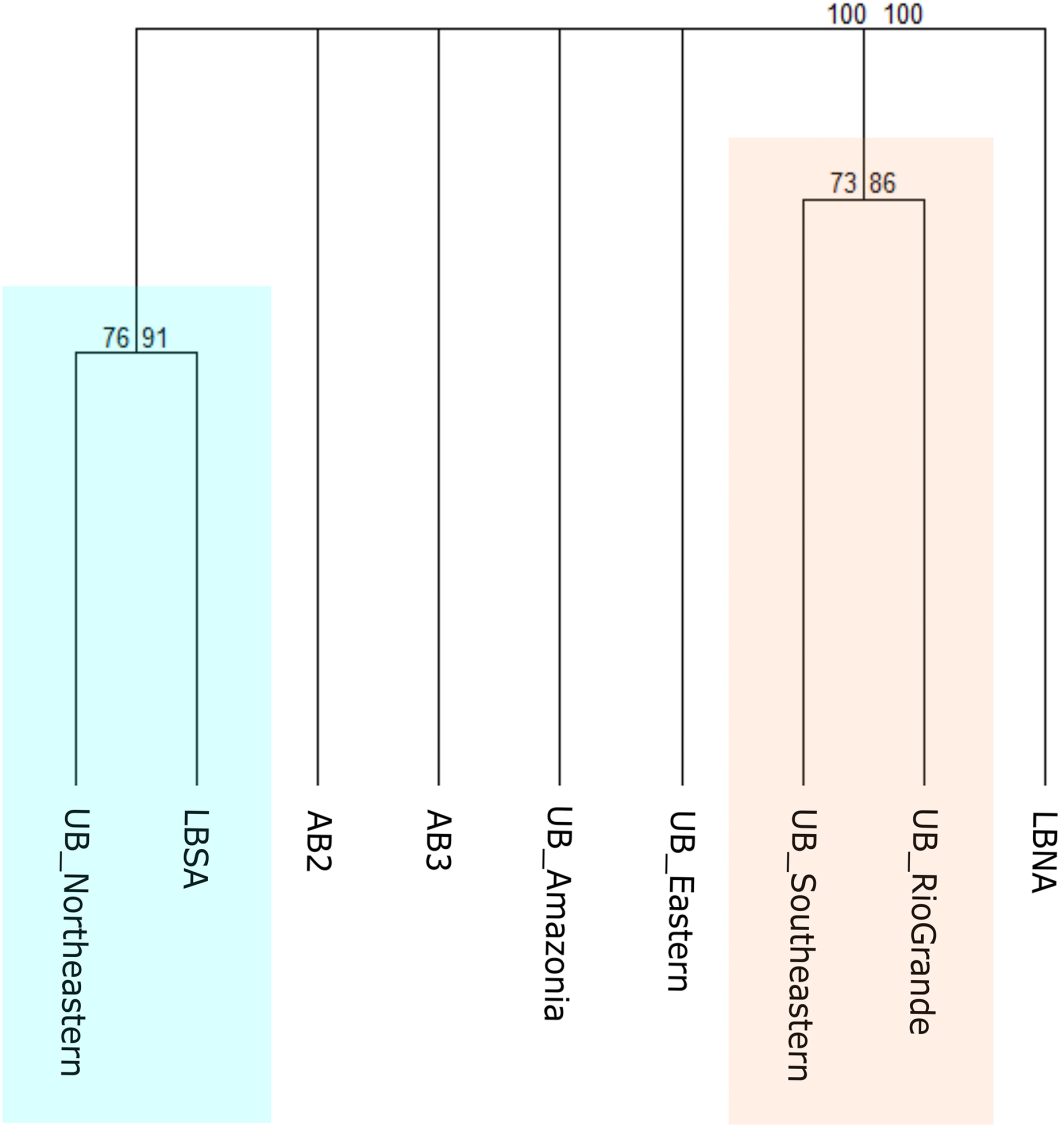
Consensus tree exhibiting the similarity between the MEOW ecoregions and the provinces proposed by Watling et al. (2013) in terms of their benthic fauna. The consensus tree obtained by resampling (1000×) the row order and a 50% node consensus rule for faunistic beta-diversity computed with the ‘recluster’ package (Dapporto et al., 2020) is represented together with bootstrap (BP) values for two different scales of bootstrap: X1 (left) and X3 (right). Nodes with a considerable increase in support are shown in black and nodes without a constant increase in red. MEOW Ecoregions: UB_Amazonia, Upper bathyal Amazonia; UB_Northeastern, Upper bathyal Northeastern; UB_Eastern, Upper bathyal Eastern; UB_Southeastern, Upper bathyal Southeastern; UB_RioGrande, Upper bathyal Rio Grande. Watling et al. (2013) Provinces: LBNA, Lower bathyal North Atlantic; LBSA, Lower bathyal South Atlantic; AB2, Abyssal North Atlantic; AB3, Abyssal Brazil Basin. Clusters with strong bootstrap node support are highlighted in different colors.

To test this scheme, benthic records were clustered according to the MEOW ecoregions (Amazonia, Northeastern, Eastern, Southeastern, and Rio Grande) for the upper bathyal depth, and according to Watling et al. (2013) for the lower bathyal and abyssal depths (Fig. 3B). It resulted in two major geographical groups with strong bootstrap node support, that have nested individual biogeographical units (Fig. 6). We found that all five MEOW ecoregions differ in terms of deep-sea benthic species composition, with Amazonia and Eastern appearing as individual biogeographical units, and a separation between the North and South regions is being evident. At upper bathyal depths, the similarity between Southeastern (UB_Southeastern) and Rio Grande (UB_RioGrande) in terms of the benthic community is stronger when compared to the other ecoregions at similar depth (Fig. 6). This group has several species in common including the corals *Cladocora debilis*, *Flabellum apertum*, and the four main CWCs (*Enallopsammia rostrata*, *Lophelia pertusa* (=*Desmophyllum pertusum*), *Madrepora oculata*, and *Solenosmilia variabilis*), some echinoderms such as *Amphiura flexuosa*, *Ophiacantha cosmica*, *Ophiomastus satelitae*, and the genus *Ophiomisidium*, and also, polychaetes such as *Micronereides capensis*, *Kinbergonuphis* sp., *Magelona* sp., and *Nothria* sp. On the other hand, the Northeastern ecoregion (UB_Northeastern) appears clustered with the South Atlantic Lower Bathyal province (Fig. 6), sharing species that are distributed both in the upper and lower bathyal depths such as the cnidarians *Deltocyathus calcar*, *Lophelia pertusa* (=*Desmophyllum pertusum*), *Madrepora oculata*, and *Solenosmilia variabilis*. The North Atlantic Lower Bathyal and the abyssal provinces of Watling et al. (2013) appear as individual units, with no resemblance to any other province.

### Biogeographical scheme 3

#### The Brazilian deep water masses biogeographic scheme

**Figure 7:**
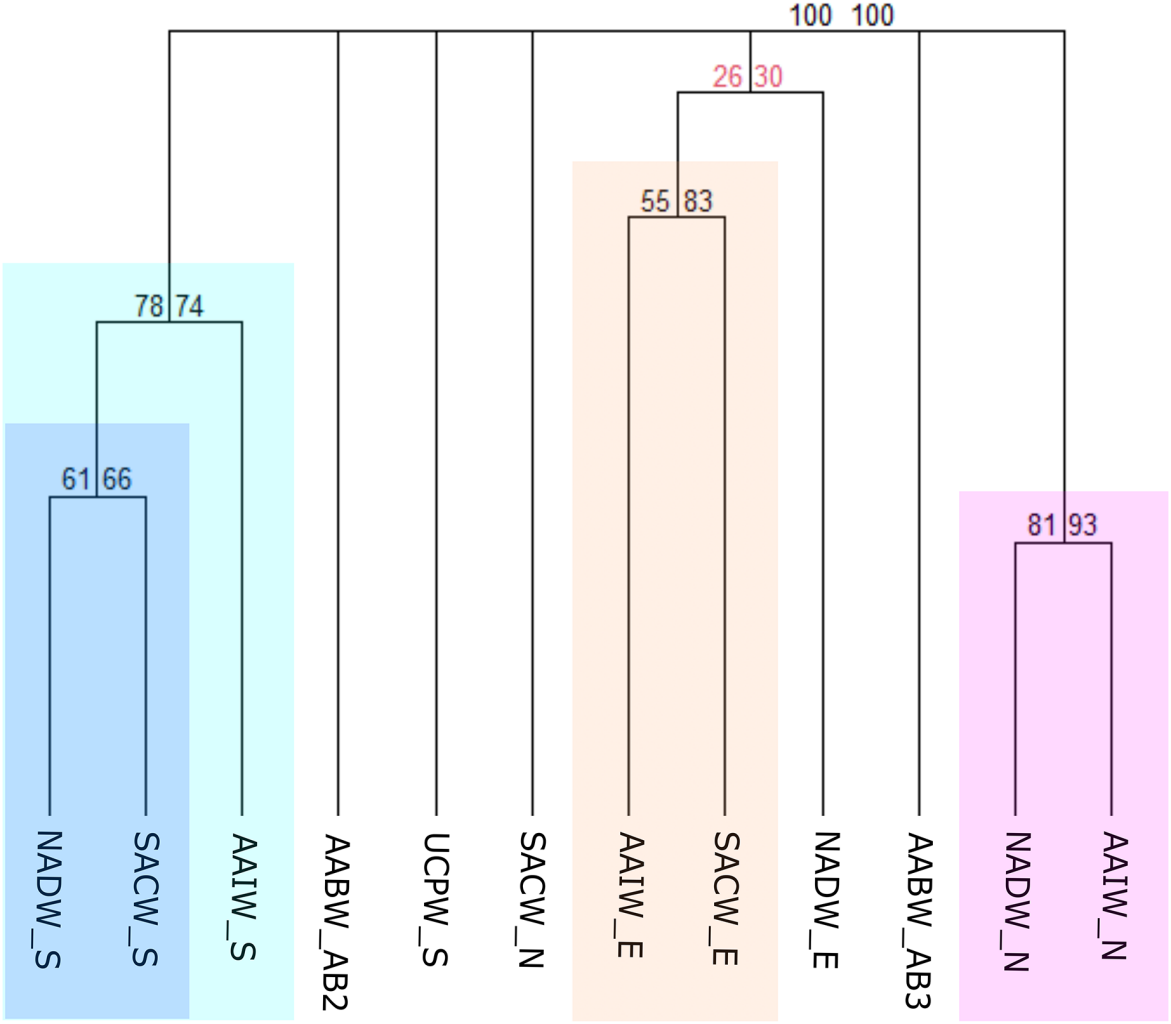
Consensus tree exhibiting the similarity between the Brazilian provinces and small units proposed in this article in terms of the deep-sea benthic assemblages of each Southwestern Atlantic water masses. The consensus tree obtained by resampling (1000×) the row order and a 50% node consensus rule for faunistic beta-diversity computed with the ‘recluster’ package (Dapporto et al., 2020) is represented together with bootstrap (BP) values for two different scales of bootstrap: X1 (left) and X3 (right). Nodes with a considerable increase in support are shown in black and nodes without a constant increase in red. The nomenclature is composed by the water mass and the region or province. N, North region; E, East region; S, South region; SACW, South Atlantic Central Water; AAIW, Antarctic Intermediate Water; UCPW, Upper Circumpolar Water; NADW, North Atlantic Deep Water; AABW, Antarctic Bottom Water; AB2, Abyssal North Atlantic; AB3, Abyssal Brazil Basin. Clusters with strong bootstrap node support are highlighted in different colors.

In this third scheme, benthic records were clustered according to the water masses flowing along the deep Brazilian margin, which was divided into North, East, and South (Fig. 3C) regions. The clustering of benthic occurrences resulted in four main biogeographic groups structured mainly by region with strong bootstrap node support (Fig. 7). The first group with the stronger node support is composed of the NADW and AAIW water masses of the North region (NADW_N and AAIW_N) that share some genera of gastropods such as *Acteon*, *Benthonella*, *Drilliola*, *Odostomia*, and *Omalogyra* (Fig. 7). A second group is formed by water masses on the East region (SACW_E and AAIW_E), supported mainly by the presence of the CWCs *Madrepora oculata, Lophelia pertusa* (=*Desmophyllum pertusum*), and *Solenosmilia variabilis*, followed by other cnidarians such as *Deltocyathus eccentricus, Cirrhipathes* sp., and *Flabellum* sp., and also by sponges of the genera *Aplysina* sp. and *Cinachyrella* sp. The NADW_E seems to be related with the last cluster, however, exhibiting a weak bootstrap node support. In the South region, the North Atlantic Deep Water (NADW_S) and the South Atlantic Central Water (SACW_S) form a distinct biogeographic group which in turn is related to the Antarctic Intermediate Water (AAIW_S) (Fig. 7). In this case, cnidarians such as *Cladocora debilis* and species of the genus *Deltocyathus* appear as shared species between the SACW and NADW; likewise, the crustacean *Parapenaeus americanus* is a common species that only presented occurrences in these two water masses. Additionally, several polychaeta genera such as *Bathyglycinde*, *Leodamas*, *Notomastus*, and *Paradiopatra* are shared between the AAIW and SACW water masses from the South. The SACW_N and the UCPW_S appear as individual units, as well as the AABW water mass (abyssal provinces AABW_AB2 and AABW_AB3), suggesting that abyssal realms have a distinct faunal composition from bathyal depths and across latitudinal gradients on the BCM (Fig. 7).

## Discussion

Here we tested the current biogeographic schemes for the deep-sea Brazilian margin and proposed new regional divisions based on benthic assemblages from bathyal and abyssal realms. We found that assemblage datasets improved the spatial delineation of previously proposed provinces or biogeographical realms, and generally support the delimitation of deep-sea boundaries based on physical-chemical characteristics of the water masses. Therefore, our hybrid scheme as well as water masses not only shows that the composition of bathyal benthic fauna actually differs between the Northern and Southern of the BCM, but also that there is a new and previously unrecognized biogeographic unit on the transition between the tropical and subtropical realms with a distinct set of species, the “Eastern”.

In general, the distribution of deep-sea bottom fauna follows the dynamics of water masses, productivity at the surface of the ocean, and climatic zonation (Vinogradova, 1979; Zezina, 1997; Carney, 2005; McClain & Hardy, 2010; McClain & Schlacher, 2015). However, in some areas the benthic distribution patterns have shown to be more related to horizontal (latitudinal), rather than vertical limits (water column) (De Leo, Bernardino & Sumida, 2020; Summers & Watling, 2021), and our results appear support those regional processes.

In the Brazilian margin, there is a marked latitudinal biogeographic delimitation by temperature that sets apart the tropical and subtropical regions. Spalding et al. (2007) proposed two realms and three provinces for the Brazilian margin. The realms correspond to the tropical and subtropical portions of the BCM, and the provinces are nested within the realms. This is also evident in the distribution of benthic records and from previous oceanographic datasets from the Atlantic (Watling et al., 2013). Deep-sea boundaries into these tropical and subtropical realms and their resulting biogeographical units suggested that water masses and ocean productivity (POC flux) were key to create two bathyal and three abyssal provinces in the deep South Atlantic (Watling et al., 2013). There, the lower bathyal provinces are described as having a mean bottom temperature difference of 3 °C between North and South, but both exhibit similar mean POC flow values and dissolved oxygen, being higher in the North with 4.92 g Corg m^−2^ yr^−1^ and 5.24 ml l^−1^, respectively (Watling et al., 2013).

However, the upper bathyal was not contemplated in the biogeographic scheme, so this made us to define limits for this depth range within the Brazilian continental margin, considering the same latitudinal limits used by Watling et al. (2013) for the lower bathyal provinces (Fig. 3B). Analyzing our results and comparing the resulting clusters from the biogeographic schemes, we agree with the boundary from Watling et al. (2013) and believe that a division of the Atlantic into two large biogeographical units (North Atlantic and South Atlantic) should be maintained under the name of “realms” following the terminology of the MEOW classification (Spalding et al., 2007).

The resulting clustering from our analysis shows that the South (Southeastern and Rio Grande) and the North (Amazonia and Northeastern) provinces are contrasting with the shallow-water MEOW boundaries, in better accordance with the limits of the Atlantic provinces proposed by Watling et al. (2013) (Fig. 6). The Southeastern and Rio Grande regions presented a higher similarity within the ecoregions analyzed in terms of the upper bathyal, where these regions form a single one together with strong node support (Fig. 6). The differences between coastal and deep-sea boundaries from the MEOW provinces arise likely due to major differences in habitat and abiotic variables (geomorphological, hydrographic, and geochemical) inside the BCM. However, even when the classification of Spalding et al. (2007) provided a good starting point for upper bathyal analyses, it is limited to coastal marine habitats and the observed deep-sea boundaries on the South Atlantic are distinct from shallow-water habitats (Aued et al., 2018).

A significant part of the Brazilian EEZ corresponds to the lower bathyal, characterized by a narrow continental slope and rapid changes in bathymetry, making it difficult to define the precise limits between upper bathyal biogeographical units. The third scheme allowed us to test the assumption of a vertical species distribution linked to depth limits in deep-sea, where the SACW of North and East regions exhibited a marked difference in benthic composition compared to the deeper water masses of each region (Fig. 7). This can be due the connectivity of both regions with a variety of shelf ecosystems and the strong influence of the Amazon plume in the north (Lana & Bernardino, 2018; Sumida, De Leo & Bernardino, 2020), which was evidenced with SACW_N as an individual biogeographical unit. In contrast, our results showed that SACW in the East is the water mass with the major number of species with 49% of total benthic species reported for the BCM and more than 80% of them are distributed in the SACW only, while NADW exhibits less than 30% of the species from the Eastern region. This high richness has been reported due to a great diversity and habitat heterogeneity (Cord et al., 2022), including several deep-sea ecosystems such as canyons, seamounts, and guyots (Bernardino et al., 2019).

In addition, the Eastern region can be considered a transition zone between the tropical and subtropical portions of the BCM, given the presence of the Vitória Trindade Ridge (Silveira, Napolitano & Farias, 2020), and the influence of coastal upwelling, being most prominent near Cabo Frio at 23°S (Valentin, 2001). The enhanced primary productivity increasing in food supply to the benthic communities can be a driving variable to increase species connectivity between the East and Southern regions of the Brazilian margin that are more productive (Watling et al., 2013). In our study, nearly 86% of the species recorded for the South region are distributed in the SACW, followed by the AAIW. Both water masses bifurcate near the boundary with the East region, the SACW bifurcates into the Vitória Trindade Seamount chain and the AAIW at 23°S, being able to transport the nutrients coming from the Cabo Frio upwelling towards the South.

In the South, the UPCW water mass showed a clear separation of the other water masses in the region (Fig. 7). Even though we had only a few records from the UPCW, most of them were unique to this water mass which has lower temperature than its surrounding water masses, but twice the content of phosphates and silicates. However, few cold-water coral records were shared with the surrounding NADW water mass from the South and East regions. The NADW, unlike the UPCW, is characterized by high oxygen and temperature values, and low local levels of nutrients (Silveira, Napolitano & Farias, 2020).

Despite having few data on the abyssal provinces, they behaved as expected. Taxonomic isolation of deep-sea fauna in different regions has been shown to increase with depth (Vinogradova, 1961; Rex, 1973; Sibuet, 1977). Both the North Atlantic Abyssal (AB2) province and the Brazilian Basin (AB3) appear as individual biogeographical units in all three biogeographic schemes (Figs. 5–7), with a few species with wide distribution in the Atlantic, including the scleractinian corals *Eguchipsammia cornucopia* and *Polycyathus senegalensis*. Although the records of the abyssal fauna for the Brazilian margin are still scarce, especially for benthic fauna, our data supports the Atlantic abyssal provinces of Watling et al. (2013) (North Atlantic, Brazilian Basin and Argentine Basin). These provinces not only differ strongly in the mean bottom temperature but also in the POC flux from North to South (Watling et al., 2013), which can be structuring the benthic provinces in these highly food-limited environments (Smith et al., 2008). In this way, the Brazilian Basin is considered an area of low to moderate abyssal POC flux, located between two provinces of moderate to high POC flux (Watling et al., 2013).

Even though the Deep South Atlantic Ocean has seen an increment in sampling effort in recent decades due to an increased interest on economic resources (Almada & Bernardino, 2017; Bernardino & Sumida, 2018; Perez et al., 2020), open biodiversity datasets from this region are largely scarce when compared to data-rich regions of the Pacific or the North Atlantic (Menegotto & Rangel, 2018; Saeedi et al., 2019a). This limited number of biodiversity records in the deep South Atlantic likely arise from both lower exploration and sampling effort, but also from historical research practices of not sharing data openly (Saeedi et al., 2019b; Hughes et al., 2021). In the deep-sea Brazilian margin, sampling effort has been historically driven by the offshore oil and gas industry which funded most ecological and biological studies to date (Almada & Bernardino, 2017). However, in addition to spatial and temporal under sampling, there are other taxonomic and ecological gaps along the Brazilian margin that would need to be addressed and which would be extremely valuable for a more detailed recognition of its deep biogeographic boundaries.

Although it is believed that each benthic taxonomic group may differ in its vertical and horizontal distribution patterns given its own features towards topographical barriers or environmental conditions, our attempt to compare the biogeographical results using all available benthic data for the BCM with the result using Cnidaria or Mollusca only, did not show significant differences. Furthermore, the species-level approach was expected to allow a better construction of our dendrograms, given the importance of endemic species to delimit regions, but agreeing with other biogeographical studies (Costello et al., 2017), our result do not differ from results at genus-level approach.

At greater depths, the differences between the physical characteristics of water masses are much lower, and food supply constitutes an important factor related to the distribution of benthic animals, especially macro and meiofauna (Smith et al., 2008). Our results showed that the distribution of deep-sea benthic fauna in the Brazilian EEZ seems to be driven mostly by latitudinal boundaries rather than water masses. Notwithstanding, some water masses showed singular features such as SACW and UPCW, which exhibit a very different species composition than their adjacent water masses, but more data will be needed to draw conclusions.

**Figure 8:**
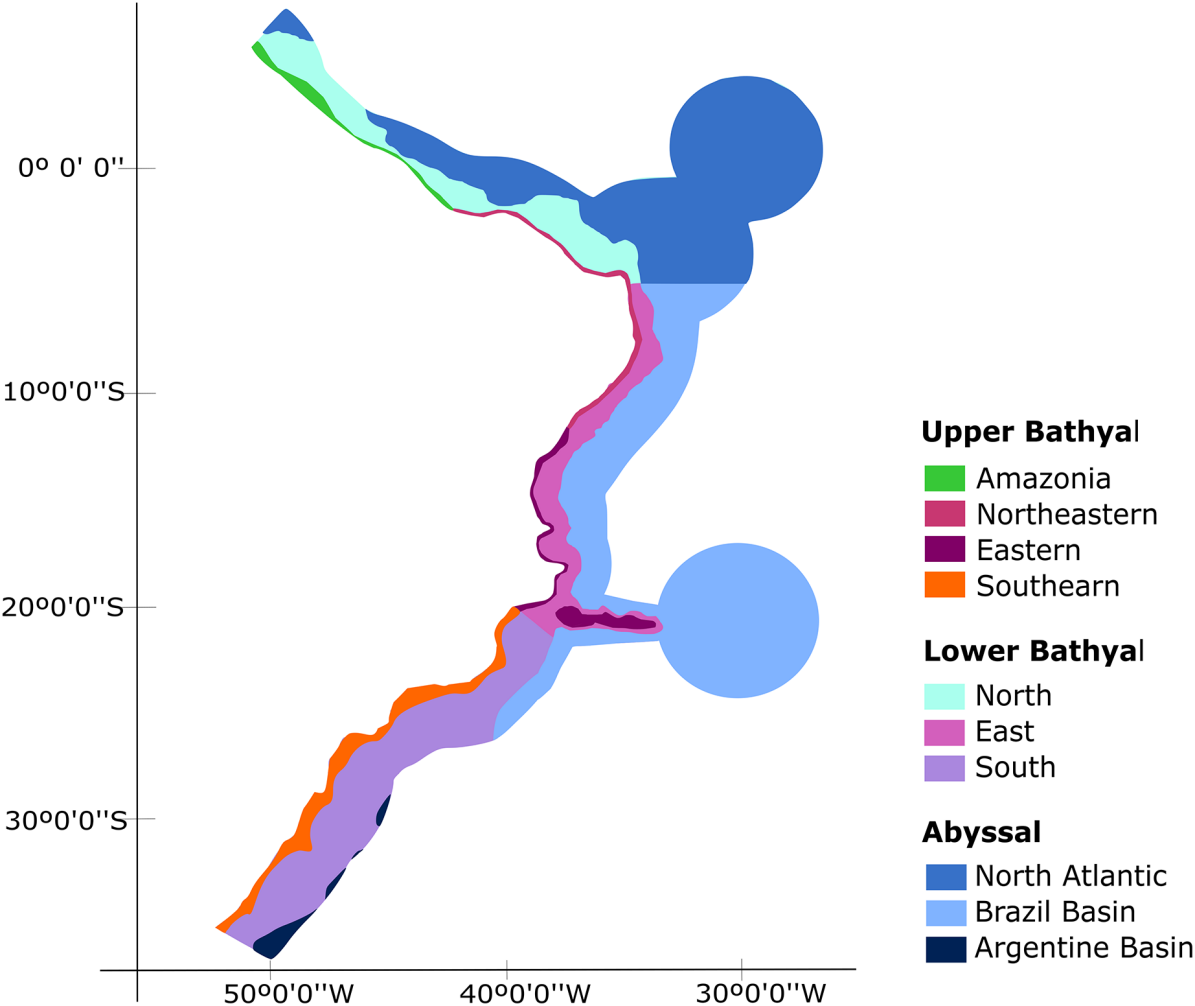
Biogeographic proposal for the deep Brazilian margin.

Our biogeographic analyses support the bathyal South Atlantic deep-sea provinces proposed by Watling et al. (2013) but suggest marked regional differences in upper bathyal ecoregions that were not previously recognized from deep-sea oceanographic patterns. We suggest that the BCM is biogeographically separated into four deep-sea ecoregions along its upper bathyal depths (200–1,000 m depth; Fig. 8). These include deep Amazonia, Northeastern, Eastern and Southern regions. On the other hand, at lower bathyal depths (1,000–3,500 m depth), we propose the use of three deep ecoregions, which include a North, East, and South region with latitudinal limits at 4°N-5°S, 5°S-21°S and 21°S-35°S, respectively. Finally, we propose the maintenance of the three provinces of Watling et al. (2013) for the Abyssal Atlantic.

## Conclusions

In this study we updated existing biogeographic classifications for the deep South Atlantic along the Brazilian continental margin. Our study revealed new latitudinal limits along the bathyal depths of the BCM with a new and formerly unrecognized biogeographical unit on the transition between the tropical and subtropical realms. Our study supports that the broad-scale distribution of benthic fauna in the Brazilian deep sea has a strong relationship with water masses characteristics. Specifically, there is a strong division between the upper bathyal (200–1,000 m), lower bathyal (1,000–3,500 m) and abyssal (>3,500 m) depths, which constitute three different environments, each one with their particular faunal composition, but sharing some species between them. In addition, in some areas of the BCM, benthic distribution patterns appear to be more related to horizontal than vertical boundaries. Some of these latitudinal boundaries remain directly related to changes in water column composition that may be influenced by changes in seafloor geomorphology. Furthermore, our work highlights the need for more species distribution studies in the deep-sea, for future comparisons between distribution patterns that allow adequate planning of conservation and protection areas for deep benthos.

## Additional Information and Declarations

### Data Availability

The following information was supplied regarding data availability: The findings of this study are available in PANGAEA Data Publisher for Earth & Environmental Science: Gaurisas, Daniela Y.; Bernardino, Angelo Fraga (2022): Benthic biodiversity and biogeographic patterns on the Deep Brazilian Margin. PANGAEA, https://doi.org/10.1594/PANGAEA.950760.
